# Evaluation of Homocysteine Levels in Patients with Polycystic Ovarian Syndrome 

**Published:** 2011-02-20

**Authors:** Saghar Salehpour, Ozra Manzor-al-ajdad, Elham Neisani Samani, Alireza Abadi

**Affiliations:** 1 Department of Obstetric and Gynecology, Infertility and Reproductive Health Research Center (IRHRC), Shahid Beheshti University (Mc.S), Tehran, Iran

**Keywords:** Polycystic Ovary Syndrome, Homocysteine Blood, Cardiovascular Disease

## Abstract

**Background:**

To determine the level of plasma homocysteine in patients with polycystic ovary syndrome (PCOS) compared with healthy controls.

**Materials and Methods:**

In this prospective case-control study on 85 PCOS women and 83 controls matched by body mass index (BMI), homocysteine levels were assessed.

**Results:**

The mean level of homocysteine was 16.25 ± 11.94 μmol/L in patients with PCOS and 11.58 ± 3.82 μmol/L in controls (p=0.002). Patients with PCOS had a significantly higher risk for hyperhomocysteinemia compared with BMI-matched control women.

**Conclusion:**

These data suggest that homocysteine levels are elevated in the PCOS population. Further studies are needed to characterize this relationship.

## Introduction

Polycystic ovary syndrome (PCOS) is the most common endocrinological disorder amongst reproductive age women. The pathophysiology of PCOS is complex in that it causes a spectrum of manifestations (1-4). It is associated with a variety of comorbidities such as diabetes, hypertension, dyslipidemia, cardiovascular events and malignancies that manifest at a young age (4, 5). Preliminary investigations suggest that serum biomarkers of cardiovascular disease such as highsensitivity C-reactive protein, homocysteine and adiponectin are abnormal in women with PCOS (6-9). Since hyperhomocysteinemia is a risk factor for cardiovascular diseases, it has been postulated that homocysteine levels are higher in PCOS patients than controls. Homocysteine is an amino acid formed by the conversion of methionine to cysteine. It is metabolized by one of two pathways: trans-sulfuration and remethylation. This process requires vitamin B as a cofactor (5-8). Normal homocysteine concentrations range between 5 and 15 μmol/L. Hyperhomocysteinemia has been classified as follows (10): mild (15 to 30 μmol/L), intermediate (30 to 100 μmol/L) and severe (>100 μmol/L). Elevations in plasma homocysteine are common and occur in five to seven percent of the general population. Hyperhomocysteinemia is an independent risk factor for atherosclerotic vascular disease, cerebrovascular events and recurrent venous thromboembolism. It can occur due to genetic defects in the enzymes involved in homocysteine pathways such as methylene tetrahydrofolate reductase (MTHFR), to deficiencies in vitamin cofactors, or additional factors which include certain chronic medical conditions and drugs, such as fibrates and nicotinic acid (11-17). Several studies have investigated homocysteine levels in PCOS patients (18-28). Most have shown that women with PCOS have elevated homocysteine levels when compared with controls (19-24). Recently, Mancini et al. in their prospective case-control study on 44 patients have shown that homocysteine levels did not differ between PCOS and control women (18). Because of the complex limitations of these studies, such as a lack of uniformity in the definition of PCOS and information on levels of other cofactors, the results vary. The purpose of this paper is to evaluate homocysteine levels in the PCOS population compared with controls.

## Materials and Methods

Eighty five patients with PCOS who had no additional metabolic or cardiovascular diseases were enrolled in a prospective nonrandomized case-controlled clinical study. PCOS cases (n=85) and controls (n=83) were recruited from the Gynecology Clinic at Taleghani Hospital, Tehran, Iran between 2008-2009. All cases had a confirmed diagnosis of PCOS based on the Rotterdam diagnostic criteria: oligoamenorrhoea (inter-menstrual interval greater than 42 days) and hyperandrogenism which was defined clinically as a Ferriman-Gallwey score of 8 and/or the presence of androgenic alopecia, and biochemically as a serum testosterone concentration 2.36 nmol/l and/or free androgen index 8.98 (29). The cut-off value for defining hyperhomocysteinemia was 15 μmol/L. Other causes of hyperhomocysteinemia such as deficiencies in vitamins B12, B6, folic acid, and history of chronic medical conditions which included renal failure, thyroid dysfunction, liver diseases, drugs such as fibrates and nicotinic acid and smoking were previously excluded. All control women had regular menstrual cycles. Therefore, none of the control women fulfilled any of the Rotterdam diagnostic criteria for PCOS. All control women had regular menstrual cycles and normoandrogenaemia. No confounding medications (hormonal preparations, Metformin, Simvastatin and other related medications) had been taken by any of the subjects within three months of the study in both groups. A perfect match was obtained for body mass index (BMI) between PCOS cases and controls. The study was approved by the Ethics Committee of Shahid Beheshti University of Medical Science. All subjects provided fully informed consents.

Serum homocysteine (total) levels were measured using the RIA technique (LINCO Research, St. Charles, MO, USA). All blood samples were taken following an overnight fast.

For comparisons of the pair-matched groups of PCOS cases and controls, we used paired-sample t-tests. All analyses were conducted with SPSS (version 12.0 for Windows; SPSS Inc., Chicago, IL, USA). P<0.05 was considered significant.

## Results

The mean age of women was 29 ± 5.9 years in the PCOS group and 27.3 ± 5.3 years in controls (p=0.06). The mean baseline BMI was 26.7 ± 4.2 kg/m² and 25.8 ± 4.1 kg/m² in PCOS and controls, respectively (Table 1). According to the t test there were no meaningful differences between these groups in BMI (p=0.1). The mean level of homocysteine was 16.25 ± 11.94 μmol/L in patients with PCOS and 11.58 ± 3.82 μmol/L in controls.

Paired-sample comparisons revealed that patients with PCOS had a significantly higher risk for hyperhomocysteinemia when compared with BMImatched control women (Table 2).

**Table 1:** Demoghraphic data in patients with PCOS and controls


	PCOS (n=85)	Controls (n=83)

**Age**	29 ± 5.9	27.3 ± 5.3
**BMI (kg/m²)**	26.7 ± 4.2	25.8 ± 4.1
**Homocysteine level (mean ± SD) µmol/L**	16.25 ± 11.94	11.58 ± 3.82


All values are mean±SD.

**Table 2:** Comparison of hyperhomocysteinmia in PCOS patients and controls*


Hcy**(µmol/L)	≥ 15	< 15

**PCOS (n=85) **	39 (45.8%)	46 (54.1%)
**Controls (n=83) **	22 (26.5%)	61(73.4%)


* Odds ratio: 2.35, 95% CI (12.65-17.35) **Homocysteine levels

## Discussion

Our data showed that serum homocysteine levels were significantly higher in PCOS women than controls. Our findings are consistent with a previous smaller study by Battaqlia et al. (30). Morever in our review on 13 studies, with the exception of Mancini and Yilmaz, the same results were noted (20). Homocysteine has a well-known role in cardiovascular morbidity and mortality. It has primary atherogenic and prothrombotic properties. Homocysteine promotes leukocyte recruitment by upregulating monocyte chemoattractant protein-1 and interleukin-8 expression and secretion. The metabolite of homocysteine can combine with LDL-cholesterol to produce foam cells and atherosclerotic plaques. Homocysteine increases smooth muscle cell proliferation and enhances collagen production. Prothrombotic effects of homocysteine include attenuation of endothelial cell tissue plasminogen activator binding sites, activation of factor VIIa and V, inhibition of protein C and heparin sulfate, increased fibrinopeptide A and prothrombin fragments 1 and 2, increased blood viscosity, and decreased endothelial antithrombotic activity due to changes in thrombomodulin function.

Free radicals formed during the oxidation of reduced homocysteine may directly injure endothelial cells. Marked platelet aggregation may be secondary to the pro-aggregatory effects of homocysteine. Prolonged exposure of endothelial cells to homocysteine impairs the production of nitric oxide. Hyperhomocysteinemia has been linked to myocardial infarction and recurrent coronary events, adverse outcomes after angioplasty, carotid artery stenosis, recurrent venous thrombosis, osteoporosis, dementia and silent brain infarct (31, 32). The levels of homocysteine in the PCOS population compared with controls have been studied with conflicting results.

In multiple logistic regression analysis, age and BMI were not predictors of hyperhomocysteinmia. Because of the higher rate of hyperhomocysteinemia in PCOS subjects with significantly elevated fasting insulin, we suggest that it may be secondary to the higher prevalence of insulin resistance in PCOS patients (33).

Badawy et al. in their prospective case-control study on ninety PCOS women which used a cut off level of 11 μmol/L for a normal homocysteine level, found that 41.1% of PCOS patients and 2.9% of the control group had high homocysteine levels, which demonstrated the effect of insulin resistance on homocysteine levels (23). Kilic-Okman in a study of 29 patients with PCOS showed significant measures between the groups (33). In another similar study, the authors found that mean plasma homocysteine levels were significantly higher in the insulin-resistant PCOS patients as compared with non-insulin-resistant PCOS patients (34-37). Mancini et al. in their prospective case-control study on 44 patients showed that homocysteine levels did not differ among PCOS women and controls. In this study, they also assessed androgens, fasting glucose, insulin, leptin, fibrinogen, homocysteine, endothelin-1 and flow-mediated dilatation of the brachial artery to investigate their relationships to weight and PCOS. These researchers have suggested that weight and PCOS were two independent variables which have an effect on endothelial function (18). The lack of uniformity in the definition of PCOS and hyperhomocysteinemia as well as information on other cofactors such as BMI, fat distribution, and insulin sensitivity can explain the differences between our findings and other studies. To limit the potential confounding effect of disparate BMI between PCOS cases and controls, we adjusted for these differences in whole-group comparisons.

Insulin sensitivity was not assayed in this study, thus the relationship between plasma homocysteine level and insulin resistance was not determined. Although it was better to limit the potential for varied serum homocysteine levels during the menstrual cycle, therefore all controls and PCOS cases with regular menstrual cycles had blood taken during the follicular phase (days 2-5) of the menstrual cycle. However this was not possible in this study due to irregular or absent menses in patients with PCOS.

## Conclusion

However, the true risk of events due to hyperhomocysteinmia in women with PCOS is unknown because of the lack of prospective data in addition to few studies published in older women who have an inconsistent diagnosis of PCOS (38-40). Therefore, prospective studies with a well-defined PCOS population are required. Screening for hyperhomocysteinemia before the use of oral contraceptive pills and diagnosis at a young age maybe beneficial. Therapeutic lifestyle changes with diet and exercise interventions and associated cardiovascular risk factors, including insulin resistance, hypertension, and dyslipidemia should be considered.
